# Contribution of Body Composition Measures to the Increased Left Ventricular Mass Index in Young Adult Black and White Females

**DOI:** 10.1155/ijhy/8274623

**Published:** 2025-03-29

**Authors:** Jordan Sill, Jessica G. Woo, Elaine M. Urbina

**Affiliations:** ^1^The Heart Institute, Cincinnati Children's Hospital Medical Center, Cincinnati, Ohio, USA; ^2^The University of Cincinnati College of Medicine, Cincinnati, Ohio, USA; ^3^Division of Biostatistics and Epidemiology, Cincinnati Children's Hospital Medical Center, Cincinnati, Ohio, USA

**Keywords:** fat mass, left ventricular hypertrophy, subcutaneous fat, systolic blood pressure, visceral fat

## Abstract

**Objective:** We aimed to determine the contribution of various types of body composition measures to the increased left ventricular mass index (LVMI) in young adult females.

**Methods:** Data from the National Growth and Health Study (NGHS), including dual-energy x-ray absorptiometry (DEXA), magnetic resonance imaging (MRI), and echocardiogram, were analyzed (*N* = 589, 54.8% Black, mean age: 24.9 ± 0.7 years). Logistic and linear regressions were conducted to assess for the contribution of fat mass (FM) and fat-free mass (FFM) by DEXA and subcutaneous abdominal adipose tissue (SAT) mass and visceral adipose tissue (VAT) volume by MRI in relation to the increased LVMI or left ventricular hypertrophy (LVH; LVMI ≥ 38.6 g/m^2.7^).

**Results:** FM (*β* ± SE: 0.025 ± 0.002, *p* < 0.01, adjusted *R*^2^ = 0.313), FFM (0.059 ± 0.003, *p* < 0.01, adjusted *R*^2^ = 0.374), SAT (0.054 ± 0.005, *p* < 0.01, adjusted *R*^2^ = 0.291), and VAT (0.194 ± 0.019, *p* < 0.01, adjusted *R*^2^ = 0.256) were each significantly associated with the increased LVMI, with FFM having the greatest association. Black race was associated with the increased LVMI in models involving individual fat mass types (0.055 ± 0.020, *p* < 0.01 for FM; 0.054 ± 0.021, *p* = 0.01 for SAT; 0.119 ± 0.021, *p* < 0.01 for VAT). In logistic models considering all mass types, FFM (OR [95% CI]: 1.62 [1.46–1.79], *p* < 0.01) and systolic blood pressure (SBP) (1.04 [1.01–1.07], *p* < 0.01) were significant contributors to LVH (area under the receiver-operator characteristic curve 0.847), and only FFM was a significant contributor in the corresponding linear regression (*β* ± SE: 0.059 ± 0.003, *p* < 0.01, adjusted *R*^2^ = 0.374).

**Conclusions:** FFM had the greatest association with LVH and LVMI, confirming previously published data. Through the use of MRI, we found that neither subtype of abdominal fat mass (SAT and VAT) better explained the variance in LVMI than FFM.

**Summary**

1. What is already known about this subject?
• Increased left ventricular mass is an independent risk factor for cardiovascular events and death.
• The effects of subcutaneous fat mass or visceral fat mass compared to fat-free mass or total fat mass on the development of left ventricular hypertrophy (LVH) in young adult females are not known.
• Racial differences in these associations are not well-described.
2. What are the new findings in your manuscript?
• Subcutaneous abdominal fat volume and visceral fat volume assessed by magnetic resonance imaging (MRI) are independently associated with increased left ventricular mass, but their respective contributions are less than those of fat-free mass and fat mass assessed by dual-energy x-ray absorptiometry (DEXA).
• Black race is associated with increased left ventricular mass in models involving individual fat mass types.
3. How might your results change the direction of research or the focus of clinical practice?
• Fat mass, subcutaneous abdominal fat, and visceral fat are modifiable risk factors associated with increased left ventricular mass to which prevention efforts should be targeted.
• Social determinants of health that may mediate the effects of race should be further investigated.

## 1. Introduction

Obesity is a risk factor for cardiovascular disease (CVD) with risk where recent data have linked obesity in the young to fatal and nonfatal cardiac events in adulthood [1, 2]. CVD prevention guidelines recommend that the diagnosis of obesity is made by measuring the body mass index (BMI)—weight in kilograms divided by height [2]. [3–5] However, the BMI does not adequately explain the CVD risk associated with obesity as it does not account for body composition.

Two methods for measuring body composition are dual-energy x-ray absorptiometry (DEXA) for fat mass (FM) and fat-free mass (FFM) and magnetic resonance imaging (MRI) for the two tissue types or abdominal adiposity—subcutaneous adipose tissue (SAT) and visceral adipose tissue (VAT, Supporting Table 1). These mass types have different metabolic and hormonal characteristics [6, 7] and have been shown to confer different CVD risks despite the significant correlation amongst them [8]. VAT, or intra-abdominal fat, has been shown to be a marker of CVD risk independent of the BMI [9] to be associated with other CV risk factors (e.g. insulin resistance and sleep apnea) independent of the BMI and to confer greater CV risk than abdominal SAT [10]. The increased risk associated with VAT compared to other markers of obesity may be related to unique metabolic properties, proinflammatory properties, or association with increased ectopic fat distribution [11].

In early adulthood, cardiovascular events are rare, so early markers of CVD, such as left ventricular hypertrophy (LVH) or increased left ventricular mass (LVM), are studied as they have been associated with CV mortality [12]. Echocardiography is the standard method to detect LVM, which is then indexed to control for differences in body size (left ventricular mass index [LVMI]). While the LVMI has been associated with higher BMI burden over time [13], previous studies have demonstrated that FFM explained more of the variance in the LVMI than FM [14]. Nevertheless, fat mass is a modifiable risk factor for the elevated LVMI and thus worthy of further investigation. The LVMI has also been associated with VAT more strongly than SAT [15]. These results suggest that the location of adiposity may be an important determinant of LVM.

There are sex and racial differences in body composition and LVM and their association. For example, women are more likely to be obese [16] and have greater FM, SAT, and VAT than young men with the same BMI and waist circumference [8]. Additionally, the effect of obesity on increased LVM is more pronounced in women than in men [17]. Various studies show that Black women, who are more likely to be obese [16], have lower VAT volumes [18] and a greater LVMI than White women [13].

Understanding the relationship between body composition and cardiovascular structure may inform targeted strategies for CVD prevention, particularly in individuals with obesity. However, the relative contributions of FFM, FM, SAT, and VAT to LVM and any potential racial differences in these associations are not well-described in a single cohort. Our aim was to utilize an existing longitudinal data set of Black and White females to determine the contribution of body composition to the increased LVMI and to evaluate any racial differences therein.

## 2. Methods

The National Heart, Lung, and Blood Institute Growth and Health Study (NGHS) is a longitudinal cohort study designed to investigate the racial differences in the development of obesity and cardiovascular risk factors from preadolescence through young adulthood in Black and White females. Participants (50% Black) were recruited in 1987 at three clinical centers (Cincinnati, OH; Richmond, CA; Washington, DC), and initial eligibility was limited to girls aged 9–10 years who were identified as (self-reported) “Black” or “White” and lived in racially concordant households [19]. Following the initial study including all three sites (visits 1–10, 1987–1996), the Cincinnati, the OH clinical site conducted an additional 7 annual visits to extend follow-up to age 27 (visits 11–17). Of the 871 participants enrolled at the Cincinnati site, 694 (80%) were followed into young adulthood and were eligible for inclusion in this analysis.

Measurements conducted in this study have been described previously [19]. Briefly, physical exams, including height, weight, waist circumferences, skinfold thickness, and blood pressure (both fourth and fifth phases), were obtained by trained examiners at each visit. Height was measured using a special-order stadiometer, and weight was measured using a Detecto Health o Meter electronic scale. Prior to taking the blood pressure, the right arm circumference was measured in order to select the appropriate blood pressure cuff size. Three blood pressure measurements were taken at 60 s intervals, with the participant in a sitting position, using a Baum desktop standard mercury sphygmomanometer. The mean of the second and third measurement is used as the participant's blood pressure for the exam. Laboratory evaluations were performed every other year by collection of a 30-mL blood specimen after participants fasted. A portion of the serum obtained from each participant was sent to the NGHS central laboratory at Johns Hopkins University for determination of high density lipoprotein cholesterol (HDL-C), total cholesterol (TC), and triglycerides (TG). Low density lipoprotein cholesterol (LDL-C) was estimated by the Friedwald equation. The other portion of serum was sent to the University of Michigan for determinations of glucose, insulin, and high sensitivity C-reactive protein (hs-CRP) [19]. Homeostasis model assessment of insulin resistance (HOMA-IR) was calculated using the following formula: fasting insulin (mg/dL) × fasting glucose (mU/L)/405.

At the Cincinnati site, echocardiograms and DEXA scans were performed annually beginning at visit 11 (∼age 20) [13]. Echocardiography was performed using the GE Vivid 7-V7916 system (GE Healthcare, Milwaukee, Wisconsin). Images lasting 3 cardiac cycles were obtained in the parasternal long-axis, parasternal short-axis, and apical 4 chamber views with the patient in the left lateral decubitus position. The left ventricular end-diastolic diameter, end-diastolic septal thickness, and end-diastolic posterior wall thickness were measured with the Digiview Image Management and Reporting System (Digisonics, Houston, Texas), and the mean of three readings was used to calculate LVM by the Devereux formula [20] which was then normalized to height (meters^2.7^) to calculate the LVMI [21]. Stroke volume and cardiac output were calculated from traced areas of the left ventricular 4-chamber view in systole and diastole. LVH was defined as LVMI > 38.6 g/m^2.7^.

DEXA scans were performed using a Hologic QDR-1000/W total body scanner. The mass measurements include the body only and exclude the head to avoid head movement errors. Subtotal (excluding the head) FM and FFM (lean tissue mass not including bone mineral content) in kilograms were indexed to height (meters [2]) to calculate the fat mass index (FMI) and fat free mass index (FFMI), respectively. Subtotal DEXA total tissue mass (body mass) is the sum of subtotal DEXA soft tissue (FM + FFM) plus bone mineral content, so the FMI and FFMI do not add up to the BMI.

MRI scans were performed at visits 16 and 17 (∼age 24 and ∼27, respectively) to assess abdominal body fat [22]. Abdominal MRI scans were performed utilizing a GE Signa LX-MR Exite platform (MR2) body coil and processed using the IDL/CCHIPS software. Each patient had an orthogonal scout image taken followed by Axial T1, Fast Dixon, 3d GRE, and dual echo FSPGR imaging. Images were taken from the top of T12 to sacrum, with the total volume defined as the region from the 3^rd^ slice to the top of the iliac crest. Manual segmentation was then performed for the Axial T1, Fast Dixon, and 3d GRE sequences to derive the total abdominal adipose tissue (TAT), the SAT, and the VAT volumes, measured in liters.

The analysis sample of 589 was selected to include those with echocardiogram data available during the last 3 visits (visits 15–17, ages 22–27), with the latest echocardiogram data used in analysis. DEXA and MRI body composition imaging from either of the last 2 study visits (visits 16–17, ages 24–27), including the latest visit where participants had both measurements, were included. This sample of 589, of which 550 also had DEXA data and 523 had MRI data available, represents 85% (589/694) of women who attended any young adulthood visits.

### 2.1. Statistical Analyses

Demographics and data on CVD risk factors are reported from Visit 16 (mean age: 24.8 years). Mean values were calculated and stratified by race, and normality was assessed. For variables that were not normally distributed, log transformations were performed. Unpaired *t*-tests were performed to assess for significant differences between Black and White participants. We recognize that race is a social construct which may have biologic implications; however, it is beyond the scope of this analysis and available data to identify the social constructs, giving rise to racial differences in the cardiac structure or body composition.

DEXA, MRI, and echocardiographic data were analyzed from each subject's last imaging visit (at ages 22–27). Logistic regression was conducted, and the area under the receiver-operator characteristic curves (AUROC) were calculated to assess the individual contribution of FM, FFM, SAT, and VAT in relation to the presence of LVH and to assess for racial differences in these contributions. Logistic regression was repeated with all mass types incorporated into one model. The covariates of interest tested in the adjusted model were race, systolic blood pressure (SBP), diastolic blood pressure (DBP), HOMA-IR, SAT, VAT, FM, and FFM. The model was then reduced to remove nonsignificant variables. Linear regression analyses were also performed to assess contributions to the LVMI of each mass type individually and repeated with all mass types incorporated into a model, adjusting for the same risk factors. To assess for racial differences, we tested the interactions between race and body composition measures in logistic and linear regression models assessing individual mass types. For all analyses, *p* ≤ 0.05 was considered significant.

### 2.2. Ethical Considerations

This research was conducted in accordance with the principles of the Helsinki Declaration and with the human subjects' understanding and written informed consent. The NGHS study was approved by the Cincinnati Children's Hospital Medical Center Institutional Review Board (IRB numbers: original studies # 88-5-12, #00-3-12; analysis protocol #2022-0314).

## 3. Results

### 3.1. Baseline Characteristics

All 589 participants in this analysis were female by study design and were 54.8% Black with a mean age of 24.9 ± 0.7 years (Table 1). The average BMI was elevated at 28.1 ± 7.6 kg/m^2^. There were significant racial differences in CV risk factors with Black participants having higher weight (*p* < 0.01), BMI (*p* < 0.01), SBP (*p* < 0.01), hs-CRP (*p* = 0.02), insulin (*p* < 0.01), and HOMA-IR (*p* < 0.01). Total cholesterol (*p* < 0.01) and triglycerides (*p* < 0.01) were higher in White participants compared with Black participants. Black participants had greater stroke volume (*p* = 0.01) and cardiac output (*p* < 0.01). Regarding body composition, Black participants had a higher average FMI and FFMI by DEXA as well as SAT and TAT by MRI (*p* < 0.01 for all). There were no significant differences in VAT by race. As expected, the measures of body composition by DEXA and MRI were correlated (Supporting Table 2). Finally, Black participants had a greater LVMI (*p* < 0.01).

### 3.2. LVMI and DEXA-Measured FFM vs. FM

**Figure 1:**
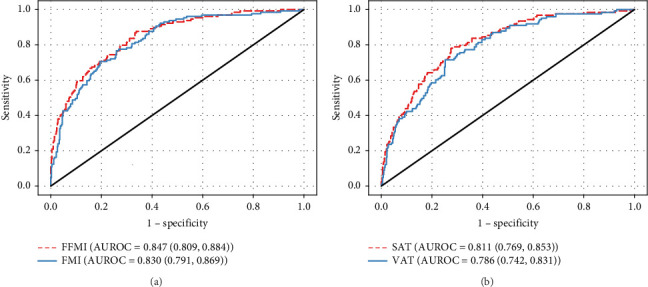
Logistic regressions of the fat mass index and fat-free mass index (DEXA) (a) and subcutaneous adipose tissue volume and visceral adipose tissue volume (MRI) (b) on left ventricular hypertrophy (LVH)⁣^∗^. ⁣^∗^All models included the race, SBP, DBP, and HOMA-IR along with the body composition parameter(s) listed.

With the FFMI and FMI analyzed individually, both retained significant associations with both LVH and LVMI (*p* < 0.01) in logistic and linear regressions, respectively. The AUROC for the FFMI was greater than the AUROC for the FMI by logistic regression (Figure 1(a)). The greater association between the FFMI versus the FMI and the LVMI was also seen by linear regression (Table 2, full models in Supporting Table 3). In both logistic regression and linear regression models including all mass types, only the FFMI remained significantly associated with LVH and LVMI (*p* < 0.01 for both).

SBP was a significant contributor in the individual FMI and FFMI logistic regressions, the individual FMI linear regression, and the combined logistic regression (*p* < 0.01 for all) but not the individual FFM linear regression or the combined linear regression. Black race was significantly associated with LVH and a greater LVMI when the FMI was analyzed individually by logistic and linear regression (*p* = 0.049 and *p* < 0.01, respectively). The remaining adjustment variables (DBP and HOMA-IR) were not significantly associated with the LVMI and LVH in these analyses.

### 3.3. LVMI and MRI-Measured Abdominal Subcutaneous vs. Visceral Fat Volume

In our individual models, both SAT and VAT were significantly associated with LVH and LVMI (*p* < 0.01 for both, Figure 1(b)). By both individual logistic and linear regressions, the contribution of SAT was greater than that of VAT (AUROC 0.811 vs. 0.786 and *R*^2^ = 0.295 and 0.261 respectively, Figure 1(b) and Table 2). In neither the combined logistic nor combined linear regressions did SAT or VAT significantly contribute to LVM. SBP was a significant contributor in the individual SAT and VAT logistic and linear regressions (*p* ≤ 0.01 for all). Black race was significantly associated with LVH in individual VAT logistic regression (*p* < 0.01) as well as a greater LVMI in the SAT (*p* = 0.01) and VAT (*p* < 0.01) individual linear regressions, but interactions between body mass types and race were not significant. The remaining adjustment variables (DBP and HOMA-IR) were not significantly associated with the LVMI and LVH in these analyses. There were no statistically significant interactions between measures of body composition and race in these models.

## 4. Discussion

Our study shows that young Black women had higher levels of CVD risk factors including BMI, SBP, hs-CRP, insulin, HOMA-IR, and measures of adiposity (FMI, SAT, and VAT). We also validated previously published reports in the NGHS cohort showing that young Black women had a higher LVMI, stroke volume, and cardiac output than Whites [13]. Consistent with previous studies, higher blood pressure was significantly associated with a greater LVMI and LVH [13, 14, 23].

We also replicated previous data showing that FFM contributes most to the variance in the LVMI [14]. This is consistent with a small adult study that used MRI to assess LVM and VAT mass (*N* = 38 participants with obesity and 16 controls with normal weight) that showed significant associations of higher FFM and VAT with a higher LVMI, with FFM having a stronger association [24].

In our analyses, the FMI lost significant association with LVH and LVMI in fully adjusted models. In a paper published by Daniels et al. including both males and females [14], the FMI remained a significant determinant of the LVMI but explained only 1.5% of the variance in the LVMI, while the FFMI explained 75% of the variance [14]. Another study using the NGHS dataset did show a relationship between higher adiposity burden over time (BMI area under the curve) with a greater LVMI [13]. However, DEXA data were not used to investigate the relationship of the LVMI with body composition parameters of FM and FFM [13].

The contributions of VAT and SAT to the LVMI in young adulthood have not been previously reported. Our analyses show that while VAT and SAT were found to be independently associated with LVH and a greater LVMI, neither significantly contributes to the fully adjusted logistic and linear regression models. This is in contrast to the study by Rider et al. which found that VAT was significantly associated with the LVMI in multivariate analysis; however, that study had a smaller and older population which also included males [24]. Additionally, our study population had lower total fat mass likely due to being recruited prior to the obesity epidemic. However, similar to our data, their model showed that over 75% of the variation in the LVMI was explained by the FFMI [24]. Other studies have shown an association between VAT and LVMI in bivariate analyses, but no adjusted analyses were reported [25, 26] or the analyses did not adjust for height or lean mass [27].

We found that there is stronger association between the LVMI and LVH with SAT than VAT. Studies with adult populations with cross-sectional MRI imaging, including the KORA S4/F4 and Dallas Heart Study, have also shown that VAT and SAT are significantly associated with increased LVM in bivariate analyses or linear regression [28, 29]. Similar to our study, these associations became nonsignificant when adjusted for other contributors [28, 29].

Our study also shows that the Black race was associated with the increased LVMI when studying the effect of visceral adiposity on the LVMI. This may relate to the lower VAT volume found in Black versus White women [18], and thus, increases in the VAT volume may reflect greater increases in other mass types or a greater proportional change in abdominal VAT. Further investigations into risk factors for the development of LVH in Black women are needed given the increased prevalence of LVH [30] and the increased risk it confers to mortality [31] in this population.

From a clinical perspective, these findings reinforce the importance of early interventions targeting modifiable risk factors. Encouraging healthy body composition during adolescence might help prevent or delay the onset of LVH and related cardiovascular issues in later adulthood. Additionally, the findings support the need for personalized risk assessment in cardiovascular care. For young adult females, body composition should be considered in routine assessments to help identify those at higher risk for LVH and later CVD.

This study has several strengths, including a large sample size in a diverse population of young women, with detailed assessment of both the cardiac structure and body composition. However, several limitations should also be considered. Due to the study design, only Black and White females who live in racially homogenous families were recruited, limiting generalizability to a more diverse population, including young men. However, this allowed for evaluation of the racial differences in the relationship between CV risk factors and cardiac structure without the confounding effects of sex. We recognize that race is a social construct rather than a biological one; however, it was beyond the scope of this paper to evaluate the specific ways that social determinants of health may have contributed to these findings [32, 33]. Future studies are needed in this area. Nearly one-third of the participants initially enrolled were not included in our analysis due to attrition which may lead to bias and limit the reliability and generalizability of our results. Additionally, as a cross-sectional analysis, this study is unable to establish causation or assess the impacts of changes in body composition over time on LVM. Our echocardiographic data were limited to measurements of the LVMI and thus did not distinguish between pathologic hypertrophy and benign hypertrophy (e.g. athlete's heart); however, the prevalence of the latter is low even amongst elite, trained athletes [34]. We utilized echocardiographic data from visits 15–17, while body composition data from DEXA and MRI were collected at only visits 16–17. Consequently, for some participants, the outcome variable (LVMI) was measured up to 2 years before the exposure (body composition). This approach was necessary due to the absence of echocardiographic data for some participants during visits 16–17. It is unlikely that substantial changes in body composition or left ventricular mass occurred during this interval, as participants were not undergoing interventions as part of the study protocol. Although our aim was to determine which measure of body composition most contributed to the LVMI, all mass types were highly correlated (Supporting Table 2), so we may be underpowered to detect independent effects. Finally, the volumes of VAT are low, which may limit the ability to discern relationships.

## 5. Conclusion

Our data show that FFM and SBP are significantly associated with the increased LVMI in Black and White young women. Additionally, FFM, FM, SAT, and VAT individually contribute to a higher LVMI, increasing the risk of developing LVH. Finally, the effect of visceral fat mass on the LVMI is influenced by the race. While the contribution of the FFMI towards the development of LVH was greater than that of other mass types, both the FMI and SBP are modifiable risk factors and thus potentially better targets for clinical intervention.

## Figures and Tables

**Table 1:** Description of the study population overall and stratified by the race at analysis baseline (visit 16).

Variable	Total (*N* = 589)	Black (*N* = 323)	White (*N* = 266)	*p* value for difference between races
Age (yr)	24.9 (0.7)	24.9 (0.7)	24.8 (0.7)	0.56

*Anthropometrics and hemodynamics*				
Height (cm)	164.4 (6.5)	164.0 (6.7)	164.9 (6.3)	0.11
Weight (kg)	76.0 (21.1)	80.8 (22.0)	70.1 (18.3)	< 0.01
BMI (kg/m^2^)	28.1 (7.6)	30.0 (7.9)	25.8 (6.6)	< 0.01
SBP (mmHg)	106.8 (10.1)	108.5 (10.2)	104.8 (9.6)	< 0.01
DBP (mmHg)	67.0 (9.8)	67.7 (10.7)	66.1 (8.7)	0.06
MAP (mmHg)	81.7 (8.8)	82.8 (9.4)	80.3 (7.7)	< 0.01
HR (bpm)	73.0 (9.9)	72.9 (9.8)	73.2 (10.0)	0.63

*DEXA body composition*				
Fat mass (kg)	23.0 (13.8)	25.5 (15.7)	20.2 (10.8)	< 0.01
Fat mass index (kg/m^2^)	9.7 (5.3)	10.9 (5.6)	8.5 (4.6)	< 0.01
Fat-free mass (kg)	44.1 (6.5)	45.3 (6.9)	42.8 (5.7)	< 0.01
Fat-free mass index (kg/m^2^)	17.1 (2.8)	17.9 (2.9)	16.2 (2.4)	< 0.01

*MRI abdominal fat*				
Subcutaneous adipose tissue (L)	3.3 (2.3)	3.8 (2.4)	2.7 (2.0)	< 0.01
Visceral adipose tissue (L)	0.6 (0.6)	0.6 (0.5)	0.7 (0.6)	0.26
Total adipose tissue (L)	3.9 (2.8)	4.4 (2.8)	3.3 (2.6)	< 0.01

*Laboratory measures*				
TC (mg/dL)	169.9 (34.9)	164.4 (32.3)	176.5 (36.8)	< 0.01
TG (mg/dL)	102.9 (81.4)	87.4 (58.2)	121.5 (99.7)	< 0.01
LDL-C (mg/dL)	99.9 (30.5)	98.3 (29.1)	101.9 (32.0)	0.17
HDL-C (mg/dL)	49.8 (11.4)	49.0 (10.9)	50.9 (12.0)	0.06
Hs-CRP (mg/L)	3.7 (5.5)	4.2 (6.2)	3.1 (4.6)	0.02
Glucose (mg/dL)	86.9 (27.6)	87.5 (29.6)	86.1 (25.0)	0.57
Insulin (microIU/mL)	11.8 (9.4)	13.7 (10.2)	9.5 (7.7)	< 0.01
HOMA-IR	2.5 (2.3)	2.9 (2.6)	2.0 (1.7)	< 0.01

*Echocardiographic measures*				
LV mass (g)	126.4 (36.1)	133.1 (36.6)	118.1 (33.7)	< 0.01
LV mass index (g/m^2.7^)	33.0 (9.3)	34.9 (9.2)	30.7 (8.9)	< 0.01
CO (L/min/m^2^)	4.8 (1.7)	5.0 (1.7)	4.5 (1.7)	< 0.01
SV (mL)	64.7 (20.4)	67.3 (20.4)	61.3 (20.0)	0.01

*Note:* Mean (standard deviation) for the study population and then stratified by race. Unpaired *t*-tests were performed to assess for racial differences in the cardiovascular risk factors listed.

Abbreviations: BMI, body mass index; CO, cardiac output; DBP, diastolic blood pressure; DEXA, dual-energy X-ray absorptiometry; HDL-C, high-density lipoprotein cholesterol; HOMA-IR, homeostatic model assessment for insulin resistance; HR, heart rate; hs-CRP, high sensitivity C-reactive protein; LDL-C, low-density lipoprotein cholesterol; LV, left ventricular; MAP, mean arterial pressure; MRI, magnetic resonance imaging; SBP, systolic blood pressure; SV, stroke volume; TC, total cholesterol; TG, total triglycerides.

**Table 2:** Reduced linear models of fat and fat-free mass types on the left ventricular mass index.

Model	*R* ^2^	Adjusted *R*^2^	Variable	Parameter estimate (SE)	*p* value
*I. Models including individual mass types*					
Fat mass index (FMI)	0.317	0.313	FMI (kg/m^2^)	0.025 (0.002)	< 0.01
			Race (white)	−0.055 (0.020)	< 0.01
			SBP (mmHg)	0.003 (0.001)	< 0.01
			Intercept	2.874 (0.116)	< 0.01
Fat-free mass index (FFMI)	0.375	0.374	FFMI (kg/m^2^)	0.059 (0.003)	< 0.01
			Intercept	2.434 (0.057)	< 0.01
Subcutaneous adipose tissue (SAT) volume	0.295	0.291	SAT (L)	0.054 (0.005)	< 0.01
			Race (White)	−0.054 (0.021)	0.01
			SBP (mmHg)	0.003 (0.001)	0.01
			Intercept	2.983 (0.122)	< 0.01
Visceral adipose tissue (VAT) volume	0.261	0.256	VAT (L)	0.194 (0.019)	< 0.01
			Race (White)	−0.119 (0.021)	< 0.01
			SBP (mmHg)	0.004 (0.001)	< 0.01
			Intercept	2.916 (0.124)	< 0.01

*II. Combined model testing of all mass types*					
All	0.375	0.374	FFMI (kg/m^2^)	0.059 (0.003)	< 0.01
			Intercept	2.434 (0.057)	< 0.01

*Note:* Generalized linear model analyses performed to assess contributions to the LVMI of each mass type individually (I) and with all mass types incorporated into a model (II), adjusting for race, systolic blood pressure, diastolic blood pressure, and homeostasis model assessment of insulin resistance. The variables included in the final models were determined by backward selection. See Supporting Table 3 for nonreduced models. *p* values correspond to *t*-tests for individual regression coefficients.

Abbreviation: SBP, systolic blood pressure.

## Data Availability

The data that support the findings of this study are available on request from the corresponding author. The data are not publicly available due to privacy or ethical restrictions.

## Supporting Information

Supporting Table S1: Methods of measuring adiposity.

Supporting Table S2: Pairwise correlations between mass types.

Supporting Table S3: Full linear models of fat and fat-free mass types on the left ventricular mass index.

Supporting File 1: The provided Standardized Reporting of Observational studies in Epidemiology (STROBE) checklist documents the manuscript's adherence to reporting guidelines for cohort studies.
