# Supplementary material for: Contribution of Body Composition Measures to the Increased Left Ventricular Mass Index in Young Adult Black and White Females
Source: Int J Hypertens. 2025 Mar 29;2025:8274623. doi: 10.1155/ijhy/8274623 (PMC11972137; doi:10.1155/ijhy/8274623)
Supplement: Supporting Information — Additional supporting information can be found online in the Supporting Information section. [file 8274623.f1.zip › Sill-BodyCompositionAndLVMassResponse-SupportingInformation (1).docx]

| **Table S1**: Methods of Measuring Adiposity ^1^ | | |
| --- | --- | --- |
| **Method** | **Strengths** | **Challenges** |
| BMI | Simple calculation using weight and height (kg/m^2^) | Does not distinguish fat mass from fat free mass |
|  | Requires minimal resources |  |
| DEXA | Distinguishes fat mass from fat free mass | Does not distinguish visceral fat from abdominal subcutaneous fat |
|  |  | Radiation Exposure |
| MRI | Distinguishes visceral fat from subcutaneous fat | Expensive |
|  | No radiation exposure | Normative data are lacking |

| **Table S2**: Pairwise Correlations between Mass Types | | | | |
| --- | --- | --- | --- | --- |
| **Mass Type** | **FFMI** | **FMI** | **SAT** | **VAT** |
| **FFMI** | - |  |  |  |
| **FMI** | 0.787 | - |  |  |
| **SAT** | 0.832 | 0.960 | - |  |
| **VAT** | 0.616 | 0.716 | 0.732 | - |
| All correlations significant p < 0.01 | | | | |

| **Table S3**: Non-Reduced Linear Models of Fat and Fat Free Mass Types on Left Ventricular Mass Index. | | | | | | |
| --- | --- | --- | --- | --- | --- | --- |
| Model | R^2^ | Adjusted R^2^ | Variable | Parameter Estimate (SE) | p-value | VIF |
| ***I. Models including Individual Mass Types*** | | | | | | |
| Fat Mass Index (FMI) | 0.314 | 0.308 | FMI (kg/m^2^) | 0.024 (0.002) | < 0.01 | 1.551 |
|  |  |  | Race (White) | -0.050 (0.021) | 0.02 | 1.104 |
|  |  |  | SBP (mmHg) | 0.002 (0.001) | 0.10 | 1.674 |
|  |  |  | DBP (mmHg) | 0.001 (0.001) | 0.29 | 1.545 |
|  |  |  | HOMA-IR | 0.015 (0.018) | 0.41 | 1.523 |
|  |  |  | Intercept | 2.881 (0.121) | < 0.01 | 0.000 |
| Fat Free Mass Index (FFMI) | 0.381 | 0.376 | FFMI (kg/m^2^) | 0.055 (0.004) | < 0.01 | 1.612 |
|  |  |  | Race (White) | -0.025 (0.020) | 0.22 | 1.128 |
|  |  |  | SBP (mmHg) | 0.000 (0.001) | 0.82 | 1.731 |
|  |  |  | DBP (mmHg) | 0.002 (0.001) | 0.13 | 1.544 |
|  |  |  | HOMA-IR | 0.004 (0.016) | 0.79 | 1.462 |
|  |  |  | Intercept | 2.346 (0.121) | < 0.01 | 0.000 |
| Subcutaneous Adipose Tissue (SAT) Volume | 0.295 | 0.288 | SAT (L) | 0.052 (0.006) | < 0.01 | 1.729 |
|  |  |  | Race (White) | -0.049 (0.021) | 0.03 | 1.110 |
|  |  |  | SBP (mmHg) | 0.002 (0.001) | 0.15 | 1.752 |
|  |  |  | DBP (mmHg) | 0.001 (0.001) | 0.34 | 1.582 |
|  |  |  | HOMA-IR | 0.008 (0.019) | 0.68 | 1.634 |
|  |  |  | Intercept | 2.981 (0.126) | < 0.01 | 0.000 |
| Visceral Adipose Tissue (VAT) Volume | 0.263 | 0.256 | VAT (L) | 0.178 (0.023) | < 0.01 | 1.556 |
|  |  |  | Race (White) | -0.107 (0.023) | < 0.01 | 1.166 |
|  |  |  | SBP (mmHg) | 0.004 (0.001) | 0.01 | 1.695 |
|  |  |  | DBP (mmHg) | 0.000 (0.001) | 0.68 | 1.597 |
|  |  |  | HOMA-IR | 0.021 (0.020) | 0.27 | 1.639 |
|  |  |  | Intercept | 2.922 (0.128) | < 0.01 | 0.000 |
| ***II. Combined Model testing All Mass Types*** | | | | | | |
| All | 0.390 | 0.380 | FMI (kg/m^2^) | 0.009 (0.007) | 0.19 | 13.488 |
|  |  |  | FFMI (kg/m^2^) | 0.051 (0.006) | <0.01 | 3.725 |
|  |  |  | SAT (L) | -0.021 (0.016) | 0.18 | 15.115 |
|  |  |  | VAT (L) | 0.049 (0.028) | 0.09 | 2.736 |
|  |  |  | Race (White) | -0.035 (0.022) | 0.11 | 1.323 |
|  |  |  | SBP (mmHg) | 0.001 (0.001) | 0.52 | 1.800 |
|  |  |  | DBP (mmHg) | 0.002 (0.001) | 0.24 | 1.593 |
|  |  |  | HOMA-IR | -0.012 (0.019) | 0.53 | 1.747 |
|  |  |  | Intercept | 2.352 (0.140) | < 0.01 | 0.000 |
| Generalized linear model analyses performed to assess contributions to LVMI of each mass type individually (I) and with all mass types incorporated into a model (II), adjusting for race, systolic blood pressure, diastolic blood pressure, homeostasis model assessment of insulin resistance. See Table 2 for reduced models.  P-values correspond to t-tests for individual regression coefficients  Abbreviations:  DBP, diastolic blood pressure; HOMA-IR, homeostasis model assessment of insulin resistance; SBP, systolic blood pressure; VIF, variance inflation factor | | | | | | |

1. Borga M, West J, Bell JD, Harvey NC, Romu T, Heymsfield SB, Dahlqvist Leinhard O. Advanced body composition assessment: From body mass index to body composition profiling. *J Investig Med*. 2018;66:1-9
